# Relationship of self-reported pain, degree of hip dysplasia, and behavioral health diagnosis

**DOI:** 10.1093/jhps/hnaf071

**Published:** 2025-12-19

**Authors:** Heather M Richard, Daryn R Strub, Kirsten Tulchin-Francis, Craig Smith, Kevin E Klingele

**Affiliations:** Pediatric Psychology Department, Nationwide Children’s Hospital, 700 Children’s Drive, Columbus, OH 43205-2664, United States; Department of Orthopedic Surgery, Nationwide Childrens Hospital, 700 Children’s Drive, Columbus, OH 43205-2664, United States; Department of Orthopedic Surgery, Nationwide Childrens Hospital, 700 Children’s Drive, Columbus, OH 43205-2664, United States; Department of Orthopedic Surgery, Nationwide Childrens Hospital, 700 Children’s Drive, Columbus, OH 43205-2664, United States; Department of Orthopedic Surgery, Nationwide Childrens Hospital, 700 Children’s Drive, Columbus, OH 43205-2664, United States

## Abstract

This retrospective analysis investigated relationships between pain levels, the extent of hip dysplasia, and behavioral health diagnoses in adolescent and adult patients diagnosed with developmental dysplasia of the hip (DDH). 105 patients (119 hips, age: 22.5 ± 0.7 yrs, body mass index (BMI): 24.8 ± 0.53) with DDH were included, focusing on demographic information, pain assessments (VAS), lateral center-edge angle (LCEA) measurements, and behavioral health data. Patients were white (92%), female (90%), and privately insured (77%). The average VAS score was moderate, 4.2 ± 2.7. No significant difference was found in pain or LCEA based on age, although higher pain was correlated with milder dysplasia (r = .254). Patients reporting severe pain had the most mild degree of dysplasia (LCEA 18.1° ± 4.9°). When analyzing those who underwent surgery, 18 patients (20/96 surgical hips) had mild pain, with an average LCEA of 13.7° ± 8.4 and 20 patients (21/96 surgical hips) had severe pain with an average LCEA of 17.8° ± 5.2°. In the nonsurgical group (*n* = 24 hips), 38% reported moderate/severe pain with mild hip dysplasia (LCEA = 17.6° ± 4.0). 74 patients received MRI imaging; labral tears were identified in 53 hips. Higher pain scores were associated with a labral tear (*P* < .05). 30% (*n* = 32) of patients had at least one behavioral health (BH) diagnosis. A trend was observed for longer duration of symptoms in patients with BH diagnosis: 26.5 ± 26.9 months versus 19.2 ± 16.0 months, *P* = .090. This study emphasizes the intricate relationships between anatomical, psychological, and socio-demographic factors in the perception of pain related to DDH, highlighting the necessity for multidisciplinary strategies in treatment planning and surgical decision making.

## INTRODUCTION

Patients often present to pediatric orthopedic clinics with primary complaints of pain. Radiographs and clinical exams are standards of practice to investigate potential sources of discomfort and subsequent treatment options.^1–3^ Determining likely causes of hip pain follows a similar rule-out process. Developmental dysplasia of the hip (DDH) is a condition in which there is insufficient bony coverage of the femoral head by the acetabulum. This can create increased stress in and around the joint, leading to pain and functional limitations.^4–6^ Hip dysplasia is seen in 1.7%–20% of the general population, with most literature reporting a 3%–5% incidence rate occurring after infancy.^5^ Research has demonstrated that surgically correcting DDH, to put the hip in a more congruent position, yields long-term benefits of preserving the joint.^7–10^

Surgical intervention, such as periacetabular osteotomy (PAO) and/or arthroscopy have become the gold standard for patients presenting with moderate to severe dysplasia (with proper indications).^7–10^ A recent study evaluated PAO results in patients with borderline dysplasia with persistent pain.^9^ These patients were noted to have failed physical therapy and injection pre-operatively, with little to no change in pain. Specific definition of ‘failed treatment,’ such as patient adherence to physical therapy, was not reported. Due to persistent complaints of pain, surgery was pursued.

Based on anatomy, it would be logical that pain would be correlated with higher degree of deformity: the lower the lateral center age angle (LCEA), the higher the pain score. In clinical practice, it has been observed that those with mild or very mild DDH report severe levels of pain and significant physical impairment. Further clinical studies are prescribed to better understand possible pathology, such as magnetic resonance imaging (MRI).^2^ Ideally, pain and deformity are aligned, and interventions are confidently initiated.

Sometimes, however, pain reports are incongruent with clinical findings. The average age of presentation for DDH is in the early teenage years as an individual's activity and growth increase.^11^ This is also a time of development when psychosocial symptoms are more likely to present, such as depression, anxiety, academic stress, social challenges, extracurricular activities, and family life.^12^ While behavioral health symptoms and hip pathology have been discussed, the potential psychosocial factors and impact on pain reports, hip deformity and treatment are not well understood.^13^ The presence of anxiety, depression, or other mental health disorders can affect pain experience and report.^14^ These are also factors that may complicate surgery decision and post-operative rehabilitation (which is beyond the scope of this study).

The purpose of this study was to evaluate the relationship between pain, degree of dysplasia, and behavioral health diagnoses. The primary aim was to validate clinical observation of pain and deformity: Patients who have greater degree of deformity report low pain scores. The study investigated orthopedic diagnosis, radiographic measurements, MRI results, pain, age of presentation, behavioral health diagnoses, prescribed psychotropic medication, and treatment. Greater understanding of pain patterns and co-morbidities in patients with DDH presenting in adolescence and adulthood will better define treatment decisions.

## MATERIALS AND METHODS

An internal Review Board-approved retrospective review was completed on patients ages 12–40 years diagnosed with DDH who presented to a large, pediatric orthopedic service between 2015 and 2023. Patients who had underlying neuromuscular, syndromic, or congenital anomalies, femoral acetabular impingement, and those without complete records were excluded. Demographics, Visual Analog Scale (VAS) pain scores, medications, and behavioral health (BH) diagnoses and treatment were collected. The VAS is a valid, reliable pain measure, and scores range from 0–10, with higher scores indicating higher perceived pain^15^. Scores reported between 1 and 3 were classified mild pain, 4–6 as moderate, and 7–10 as severe. Radiographs from initial DDH diagnosis were measured using lateral center-edge angle (LCEA) to define degree of dysplasia.^1–4^ Hips with a LCEA measuring < 25° were defined as ‘dysplastic’. A LCEA of 5°–15° was classified as ‘moderate’; < 5° was classified as ‘severe’. When completed as part of standard of care treatment, MRI was results were used to further identify sources of pain and hip pathology.^2–3^ Comparative means statistical analyses were completed with Univariate Analysis of Variance to identify statistical significance based on age, severity of pain, severity of dysplasia, or presence BH diagnosis, along with Pearson correlation analysis. Significance level was observed at *P* < .05.

## RESULTS

An electronic medical record (EMR) search of clinically diagnosed DDH, identified 297 patients. Following exclusion of 192 patients based on underlying diagnosis, history of congenital hip dysplasia, age, and/or insufficient radiographic or pain score follow-up, 105 patients, with 119 hips (7 bilateral) were included in the analysis. The sample primarily consisted of white (92%) females (90%), aged 10–26 years (71%), who were privately insured (77%, Table 1).

**Table 1:** Demographics of study cohort diagnosed with developmental dysplasia of the hip.

Demographics	
Sex, *n* (% of 105 Patients)	
Female	94 (90%)
Male	11 (10%)
Race, *n* (% of 105 Patients)	
Asian	3 (3%)
Bi-Racial	2 (2%)
Black or African American	3 (3%)
White	97 (92%)
Insurance, *n* (% of 105 Patients)	
Medicaid	24 (23%)
Commercial	81 (77%)
Behavioral Health, *n* = 32 pts, 30% of all Patients (% of 32 Patients)	
Depression	11 (34%)
Anxiety	21 (66%)
ADHD/ADD	12 (38%)
Suicidal Ideation	5 (16%)
Age at Presentation (% of 119 Hips)	
Mean ± SD	22.5 ± 7.3
Range	12.5, 39.7
Treatment, *n* (% of 119 Hips)	
Surgical	95 (80%)
Non-Surgical	24 (20%)

As a cohort, VAS scores averaged in the low-moderate pain range (4.2 ± 2.7), with an average LCEA of 15.2° ± 7.9° (Table 2). Patients who had mild dysplasia (75 hips [63%], LCEA:19.5° ± 2.2°) reported moderate pain (VAS: 4.6 ± 2.5), while individuals who had moderate dysplasia (34 hips [29%], LCEA: 12.1° ± 2.6°) reported mild pain scores (VAS: 3.5 ± 2.7). Among the 9 patients (10 hips) with severe dysplasia (LCEA: −6.5° ± 7.5°), 4 reported a VAS score of 0, the remaining reported values ranged from 2 to 5. Pearson correlation revealed a significant weak, negative, correlation (*r*(119) = .254, *P =* .005) as higher pain scores were associated with the less dysplastic hips (Fig. 1).

**Table 2:** Mean ± SD of patient cohorts diagnosed with developmental dysplasia of the hip with respective *P*-values for comparative analysis data variables.

	VAS Pain Score	LCEA	Duration of Pain
Cohort	4.2 ± 2.7	15.2° ± 8.0°	20.9 ± 19.3
Age Group <18 18–26 >26 * P*-value	3.5 ± 2.5 4.4 ± 2.6 4.8 ± 2.9 .067	14.2° ± 8.9° 17.0° ± 5.2° 15.0° ± 8.7° .278	18.1 ± 19.7 21.0 ± 14.8 24.4 ± 22.4 .372
Treatment Surgical Non-surgical * P*-value	4.3 ± 2.6 3.4 ± 3.0 .136	15.1° ± 8.1° 15.6° ± 7.9° .807	21.2 ± 17.1 19.5 ± 31.5 .766
MRI Labral Tear No Labral Tear * P*-value	5.1 ± 2.4 4.7 ± 2.6 .596	17.2° ± 4.4° 17.3° ± 3.9° .968	22.6 ± 19.1 13.1 ± 9.3 .042
BH BH Diagnosis No BH Diagnosis * P*-value	3.7 ± 2.6 4.3 ± 2.7 .226	12.2° ± 11.2° 16.4° ± 6.2° .011	26.5 ± 26.9 19.2 ± 16.0 .100

**Figure 1:**
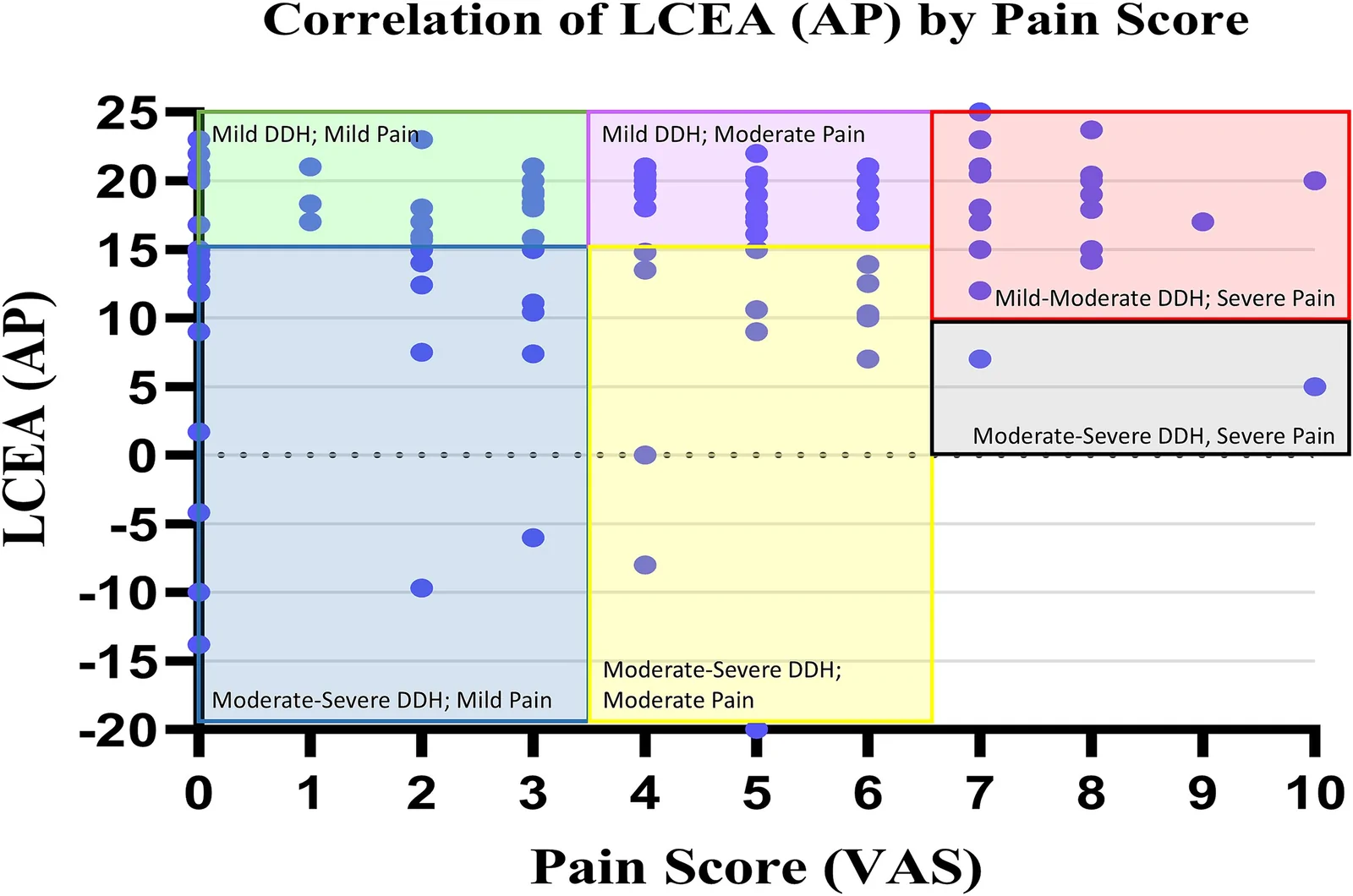
There was a weak correlation between pain scores and LCEA. Shaded regions graphically representing the division of mild, moderate, and severe pain scores and DDH.

Patients were categorized by age: individuals under 18 years, 18–26 years, and over 26 years. While there was a trend for increasing pain based on age (<18 yrs: 3.5 ± 2.5, 18–26 yrs: 4.4 ± 2.6, > 26 yrs: 4.8 ± 2.9), this was not statistically significant (*P* = .067). Similarly, there was no significant difference between groups for LCEA (<18 yrs: 14.2° ± 8.9°, 18–26 yrs: 17.0° ± 5.2°, > 26 yrs: 15.0° ± 8.7°, *P* = .278) or duration of symptoms (<18 yrs: 18.1 ± 19.7, 18–26 yrs: 21.0 ± 14.8, > 26 yrs: 24.4 ± 22.4, *P* = .372).

Eighty percent of hips (95/119) were treated surgically with a periacetabular osteotomy and/or arthroscopic labral repair. The mean LCEA in the surgical group was 15.1° ± 8.1°, with moderate reported pain (VAS = 4.3 ± 2.6, range 0–10). Similar to the entire cohort, the surgical group showed a correlation of higher reported pain with less dysplasia (*r*(95) = .248, *P =* .015). There was not a statistical difference in LCEA across pain classification within the surgical group (*n* = 14 [15%] no pain: 12° ± 9.3° versus *n* = 20 [21%] mild pain: 13.7° ± 8.8° versus *n* = 40 [42%] moderate pain: 15.5° ± 8.4° versus *n* = 21 [22%] severe pain: 17.8° ± 5.2°, *P* = .160). Similar to the surgical group, there was a non-significant, weak correlation (r(24) = .298, *P* = .157) with higher pain scores associated with less dysplasia in the non-surgical group. Eleven (46%) patients in the non-surgical group reported moderate to severe pain scores, with 6 (25%) reporting no pain.

MRI was ordered in 74 hips to further evaluate ligament and tissue integrity. Labral tears were identified in 53 hips (70%), 46 of the 53 went on to surgery. Utilizing MRI imaging, patients identified with a labral tear had higher VAS scores compared to those without labral tear (VAS = 5.1 ± 2.4 versus 4.7 ± 2.6, respectively), with an average LCEA of 17.2° ± 4.4° *versus* 17.3° ± 3.9°. A statistically significant difference in reported pain was observed between the surgical patients who had a labral tear and those who did not (VAS = 5.2 ± 2.3 versus 3.6 ± 2.7, respectively, *P* = .002), while LCEA was not statistically significant for surgical patients with/without labral tears (LCEA = 17.2° ± 4.5° versus 17.9° ± 4.2°, respectively, *P* = .609). Non-surgical patients with a labral tear reported a VAS of 4.3 ± 3.1 and those without a labral tear reported 3.1 ± 3.0, which was found not statistically significant (*P* = .380). Although not statistically significant (*P* = .428), LCEA in the non-surgical patients with a labral tear was higher (17.6° ± 3.8°) than the non-surgical patients without a labral tear (16.0° ± 3.2°).

Thirty-two patients (30%) had at least one reported BH diagnosis, with many patients reporting multiple diagnoses. Overall, the highest reported diagnoses were anxiety disorders 66% (*n* = 21), Attention Deficit Hyperactivity Disorder (ADHD) 38% (*n* = 12) and depression 34% (*n* = 11), with 16% (*n* = 5) having a history of suicidal ideation. Of patients with a BH diagnosis, 72% (*n* = 23) were prescribed psychotropic medication(s). There was no significant difference in VAS pain scores between patients with and without a behavioral health diagnosis (VAS = 3.7 ± 2.6 versus 4.3 ± 2.7, respectively, *P* = .226). Patients with a report of suicidal ideation reported higher pain (VAS = 6; Range: 2–8), with mild DDH (LCEA average of 18.4° ± 4.7°). Patients diagnosed with ADHD had the most severe dysplasia (LCEA = 8.6° ± 13.2°), but also the lowest average VAS score of 3.1 ± 3.0. A significant difference was observed in LCEA between patients with/without BH diagnoses (12.2° ± 11.2° versus 16.4° ± 6.2°, respectively, *P* = .012). Patients diagnosed BH issues reported an average symptom duration of 26.5 ± 26.9 months compared to those without BH with 19.2 ± 16.9 months (*P* = .090). While not significant, the longer symptom duration in BH patients may suggest further implications for behavioral health. Additionally, individuals with suicidal ideation had an average symptom duration of 24.8 ± 16.3 months.

## DISCUSSION

This study evaluated pain reports, BH diagnoses and treatment course in patients with DDH. This cohort primarily consisted of white, privately insured females who presented for evaluation of hip pain. In this study, higher VAS pain scores were correlated with less severe DDH. This finding was consistent within surgical and non-surgical group analyses. Of the 62% of patients who underwent MRI, 71% were diagnosed with labral tear (*n* = 53), however, not all patients went on to have surgery. It is unclear, given methodology, what other factors may have contributed.

While behavioral health and psychosocial factors are important considerations as part of treatment planning, having a known diagnosis listed in the EMR did not significantly elevate pain scores in this group. These are patients who have known diagnosis and are likely in the care of a mental health provider. Those with an identified diagnosis may have well controlled symptom management. In this study, those with a behavioral health diagnosis had more severe dysplasia, but no difference in pain scores. Of note, many of the patients in this cohort were reported to have prescription for at least one psychotropic medication. Those patients with a reported history of suicidal ideation experienced higher pain, even with mild DDH. Although not statistically significant, there was a trend for longer symptom duration in patients with a BH diagnosis, which should be considered.

The VAS pain scores were similar in both the operative and nonoperative groups. Surgical indications in hip preservation are multifactorial and while pain is certainly highly considered, there are other signs/symptoms that lead to the decision for surgery. Our study was limited to only evaluating pain as a preoperative symptom and this limits our ability to evaluate the complexity of symptoms in patients with hip pain. Patients and their families are also involved in the overall management and shared decision-making which can greatly affect the ultimate treatment plan. The perception of surgery as a comprehensive remedy for various health conditions may have fostered a growing preference for surgical interventions, potentially shaping the decisions of patients and their families in collaboration with healthcare providers. This strong belief in the effectiveness of surgical treatment can contribute to an increased rate of surgical procedures being undertaken, despite the availability of conservative treatment alternatives. In the current study, pain scores were recorded pre-operatively at a single time point during a clinic visit and do not allow us to evaluate chronicity, persistence, or the amount of functional interference. In patients with long standing dysplasia, there may be adaptive changes that take place affecting their perception of pain and function.

The goals of hip preservation surgery are to reduce pain, improve function, and prolong the natural history of the native hip. Studies have shown the more severe the dysplasia, the higher the risk of developing osteoarthritis.^5^ This increased risk of arthritis starts at LCEA < 25° and continues to increase as LCEA decreases. The PAO has been shown to improve native hip survivability. Although prophylactic PAO is still not recommended due to the unpredictable timing of symptom development and arthritis, surgeons may have a lower threshold to recommend surgical management in patients with more severe dysplasia or in those who repeatedly present to clinic. There is also more evidence that patients with mild dysplasia may have concomitant femoroacetabular impingement which could be a confounding factor contributing to their higher reported pain scores.

There are inherent limitations to retrospective chart review methodology. The data is presumed to be accurately entered into the EMR and does not account for what is not in the medical record. For example, it is possible the number of patients diagnosed with a behavioral health condition is greater, but treatment is documented in an outside record. Pain scores are recorded at each clinic appointment. Functional limitations at the time of the initial visit were not consistently noted and could not be included in this analysis. It can also not be determined if the level of physical impairment is correlated to pain, degree of dysplasia and/or other psychosocial factors.

Patient demographics are consistent with other studies discussing hip dysplasia which include primarily white, privately insured females.^4–6^^,^^9^ It is an interesting question to consider the true prevalence and psychosocial contexts inherent to this homogenous, higher socioeconomic status group.^4–6^^,^^11^ Given that pain scores in this study are lowest among those with severe deformity, what is there to say about those in more diverse populations who do not have access to an orthopedic surgeon who treats hip deformities? Perhaps, severe deformity goes undetected in minority groups and function is mildly impacted. Social determinates of health, behavioral health, context of pain score at one time point,^14–16^ and expectation of functional level, all impact the perception of pain and function,^14^^,^^16^ which provides data to determine treatment decisions. Patient expectations are also a factor in shared decision making when there are treatment options available.^17^

Prospective studies using patient reported outcome measures to evaluate pain at different time points, adherence to conservative treatments (i.e. physical therapy, medication adherence, limiting stress on the joint; intra-articular injection); mood and anxiety symptoms; goals of treatment and functional levels are indicated. Evaluating diverse groups longitudinally in terms of presence of DDH, pain, and function will also be important.

An integrated, multi-disciplinary team of surgeons, psychologists, physical therapists, athletic trainers to evaluate and intervene in a stepwise approach to manage pain and improve function will better inform the right surgery on the right patient at the right time. Determining when to surgically intervene to protect the joint may not be the same decision as when to operate for pain management or patient readiness.

## Data Availability

The data underlying this article will be shared on reasonable request to the corresponding author.
